# Annotations

**Published:** 1899-12-30

**Authors:** 


### Annotations.

### The Prince of Wales's Hospital Fund.

The report of the meeting of the General Council of the Prince of Wales's Hospital Fund, which we publish in another column, shows what a good work is being done not only by the Prince, to whose sanction and continued support the whole scheme is due, and by the general public, by whose liberality the Fund is being maintained, but also and perhaps especially by the Visitors and Distribution Committee, to whom was delegated the responsible duty of deciding which hospitals should receive of the bounty of the Fund, and the often ungrateful task of determining which institutions should be left out. Both these duties have been carefully performed, and it is very satisfactory to see that the com-

ANNOTATIONS—*continued.*

mittee have found it practicable to lay down conditions with their gifts and to give reasons for their refusals. In this way the Fund is likely, perhaps, to do even more good to hospitals taken as a whole than could be effected by the actual money gift presented to some among them. There are still some people who are ready to say that the Prince of Wales's Fund only gives to hospitals with the one hand what it has taken from them with the other, and, in fact, that the proceeding resembles that of the Paddy who is said to have cut a piece off the top of his blanket with which to keep his feet warm. We do not agree with them, but there they are. Even these objectors, however, must admit that the Prince of Wales's Fund does something more than merely give, and that the money which it distributes has an influence for good far beyond so many separate annual subscriptions. The Distribution Committee does not merely give to the hardest beggar. It gives with judgment and after inquiry—a judgment and an inquiry which the individual subscriber would find it hard to make—and it gives on a system, and thus exercises a wholesome control over the vagaries of some hospital managers. There are thousands of people who are interested from one cause and another in certain hospitals. These people, who have a local interest, and either by their vote or personal service exercise some form of control over the institutions they maintain, are the most useful givers and the best supporters of our hospitals. But there are also thousands who, while ready to give, have no personal concern in any of the numerous charities they see around them. To these the Prince's Fund appeals, and in gaining these subscriptions it does a double good—it gets money for the hospitals which would otherwise go astray, and it makes that money useful not merely in paying butchers' bills, but in encouraging careful management.

### Medical Education in London.

The question is being anxiously discussed as to what is to be done in regard to what are called the "earlier studies" for the medical degrees of the newly reconstituted University of London. While it is universally admitted that the clinical and more definitely specialised part of the medical graduate's education must be the exclusive work of the hospitals and their attached medical schools, a wish is felt that the University itself should take an active part in the teaching of such earlier and more general subjects as biology, chemistry, physics, anatomy, and physiology. With this object there are two schemes in the air; one to provide a great central University School, the other to establish, in connection with three or four of the larger existing medical schools, university institutes, where these subjects shall be taught on a University basis by University professors. The first is a scheme which, we think, might prosper, and might become the beginning of a complete severance of the earlier from the later studies, and a concentration of the former in an institute where they could be more effectually provided for than they are at present, and this not for the university students alone, but for all those working for the various qualifications. The second scheme is one which we feel confident can never come to anything; the great and insuperable objection to it being that it would at once divide the medical schools into two sets—university and non-university schools. No doubt it offers many attractions to the authorities of those schools which would be invited to sit at the cross table, but we need hardly say that it would be looked at with a jaundiced eye by those left below the salt. Nothing but jealousy and heart-burning would be raised by any attempt to put such a plan in force. Perhaps a time may come when, instead of a student who enters at a medical school being tied down by fees and custom to take from that school all the teaching he requires, whether it be good, bad, or indifferent, he will be allowed to exercise some choice, following, perhaps, the course of some famous physician at one hospital, and some equally famous teacher of surgery at another. The difficulties of organising such a scheme would be great, perhaps insuperable. Still, the advantages would in many ways be greater still, for no one can doubt that the present complete isolation of school from school is fraught with many evils, and leads to great narrowness of view. A beginning, at any rate, would be made if the earlier studies for all students were centralised in a great University school. But that, we fear, is not what is contemplated. The existing schools have to be considered. They have cost money, and they have got to be "used up." That is the key to a good deal.

### Salvation Army Shelters.

The London County Council is to be congratulated on the decision arrived at by the Court of Queen's Bench in regard to the "shelters" of the Salvation Army, namely, that they are to be looked upon as common lodging-houses and to be held subject to the conditions laid down in the Common Lodging Houses Acts. It will be remembered that in a previous case—that of Booth v. Ferret in 1890—the late Lord Chief Justice Coleridge and Mr. Justice Mathew had decided that the shelter was not a common lodging-house within the statutes on two grounds, first because it was conducted for philanthropic and charitable purposes, which these judges held took it out of the operation of the statutes dealing with common lodging-houses; and second, because everybody could not obtain admittance; and much of the interest of the present case lies in the fact that the judgment now given definitely lays it down that the case of Booth v. Ferret, on which so much has been made to hinge, was wrongly decided on both these points. This decision is one of the greatest possible importance in regard to the sanitary administration of the metropolis. It has for years been a scandal that the plea of philanthropy should be allowed to override all considerations of health, and that the Acts passed with the definite object of exercising a direct sanitary control over the lodging-houses occupied by the homeless and more or less vagrant classes should be set at defiance by people who chose to say that their particular lodging-houses were run for charity and not for gain. It has to be recognised that the most dangerous classes, so far as regards the spread of infectious disease, are the wandering and homeless ones, and that however desirable it may be to hold out to them a helping hand it is absolutely essential that in so doing we shall not set up in our midst an Alsatia in which the sanitary statutes shall be of no effect.

